# Inhibition of inflammatory microglia by dietary fiber and short-chain fatty acids

**DOI:** 10.1038/s41598-022-27086-x

**Published:** 2023-02-16

**Authors:** Maria Elisa Caetano-Silva, Laurie Rund, Noah T. Hutchinson, Jeffrey A. Woods, Andrew J. Steelman, Rodney W. Johnson

**Affiliations:** 1 Department of Animal Sciences, University of Illinois at Urbana-Champaign, Urbana, IL USA; 2 Division of Nutritional Sciences, University of Illinois at Urbana-Champaign, Urbana, IL USA; 3 Department of Kinesiology and Community Health, University of Illinois at Urbana-Champaign, Urbana, IL USA

**Keywords:** Chronic inflammation, Neuroimmunology, Epigenetics

## Abstract

Microglia play a vital role maintaining brain homeostasis but can also cause persistent neuroinflammation. Short-chain fatty acids (SCFAs) produced by the intestinal microbiota have been suggested to regulate microglia inflammation indirectly by signaling through the gut-brain axis or directly by reaching the brain. The present work evaluated the anti-inflammatory effects of SCFAs on lipopolysaccharide (LPS)-stimulated microglia from mice fed inulin, a soluble fiber that is fermented by intestinal microbiota to produce SCFAs in vivo, and SCFAs applied to primary microglia in vitro. Feeding mice inulin increased SCFAs in the cecum and in plasma collected from the hepatic portal vein. Microglia isolated from mice fed inulin and stimulated with LPS in vitro secreted less tumor necrosis factor α (TNF-α) compared to microglia from mice not given inulin. Additionally, when mice were fed inulin and injected i.p with LPS, the ex vivo secretion of TNF-α by isolated microglia was lower than that secreted by microglia from mice not fed inulin and injected with LPS. Similarly, in vitro treatment of primary microglia with acetate and butyrate either alone or in combination downregulated microglia cytokine production with the effects being additive. SCFAs reduced histone deacetylase activity and nuclear factor-κB nuclear translocation after LPS treatment in vitro. Whereas microglia expression of SCFA receptors *Ffar2* or *Ffar3* was not detected by single-cell RNA sequencing analysis, the SCFA transporters *Mct1* and *Mct4* were. Nevertheless, inhibiting monocarboxylate transporters on primary microglia did not interfere with the anti-inflammatory effects of SCFAs, suggesting that if SCFAs produced in the gut regulate microglia directly it is likely through an epigenetic mechanism following diffusion.

## Introduction

Microglia are vital for CNS development and homeostasis, and as the first immune responders in the brain, are activated by inflammatory stimuli^1^. Activation can lead to neuroinflammation to promote homeostasis, but if unchecked, activation can be pathological. Therefore, regulation of microglial cells is of utmost importance. Regulatory factors produced within the brain including interleukin (IL)-10, IL-4, and brain-derived neurotrophic factor are important, but factors produced peripherally such as corticosteroid hormones and bioactive molecules originating from the gastrointestinal system also play a role. In this context, the host microbiota and the compounds produced by microorganisms in the gut have been shown to regulate brain microglia, showing the possibility to be manipulate in non-invasive ways^2^.

Microbiota ferment dietary fiber and resistant starch in the colon, generating the short-chain fatty acids (SCFAs) acetate, butyrate, and propionate. SCFAs act locally to maintain gut health by preserving intestinal barrier integrity and promoting mucus production. Most SCFA are taken up by colonocytes using monocarboxylate transporters (MCT), and utilized as an energy substrate^3^. However, they can affect disparate physiologic systems by signaling through free-fatty acid receptor (FFAR)2 and FFAR3 (i.e., GPR43 and GPR41, respectively) on enteroendocrine cells^4^, vagal afferent nerves^5^, and immune cells^6^. They also can enter the circulatory system, cross the blood–brain barrier^3,7^, and accumulate in the brain. Indeed mice supplemented with *Clostridium butyricum*, a probiotic producer of butyrate, had higher butyrate levels in brain than in peripheral blood^8^. Acetate supplementation can increase brain acetate levels and its metabolically active intermediate acetyl-CoA, which can increase histone acetylation and suppress IL-1β expression in a rat model of neuroinflammation^9^. Further, monocytic cell lines treated with individual SCFAs showed decreased secretion of inflammatory mediators when stimulated with LPS and interferon-gamma^10^. Collectively, the literature suggests that the gut-brain axis regulates microglia through SCFAs, but the mechanisms by which this occurs remain elusive. Therefore, in the present study we hypothesized that feeding a diet supplemented with soluble fiber would increase SCFAs and inhibit inflammatory microglia. We further hypothesized that SCFAs would act directly on microglia to regulate inflammatory response. The important findings show that feeding a diet with soluble fiber increases SCFAs in cecum and hepatic portal blood and suggest that SCFAs reduce microglia activation by targeting the NF-κB pathway using a mechanism not dependent on MCTs or known receptors for SCFAs.

## Results

### Dietary fiber increased SCFA production and reduced inflammatory response of microglia

**Figure 1:**
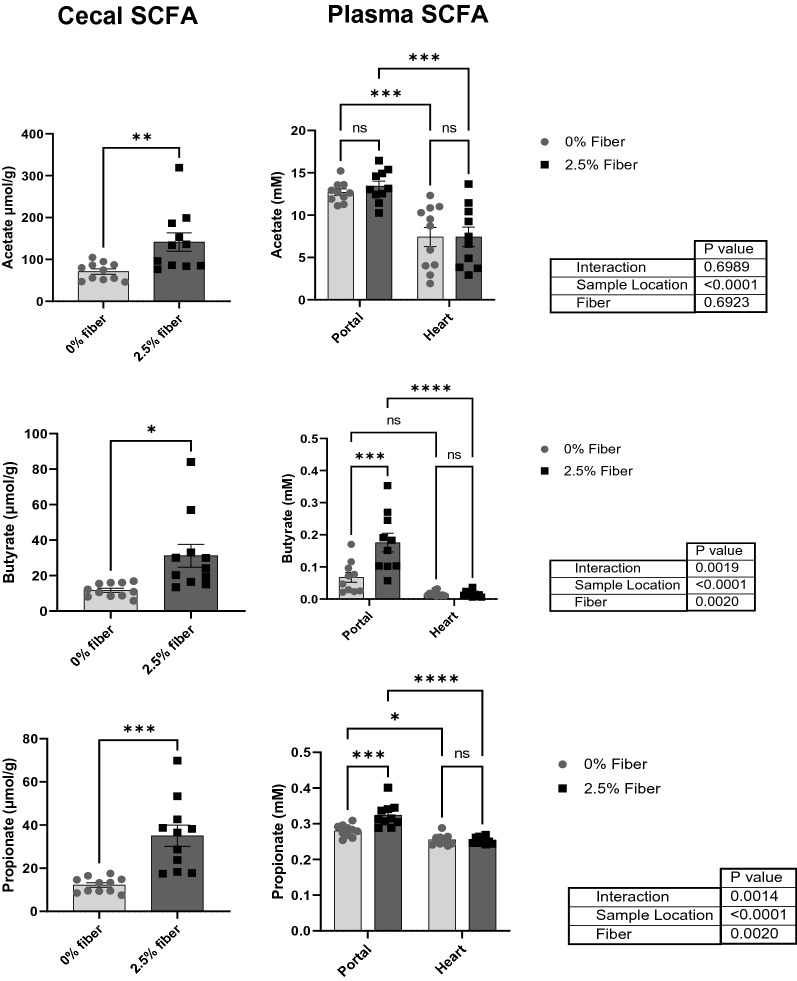
Effect of dietary fiber intake on the SCFAs (acetate, butyrate, and propionate) levels in the cecum, portal vein plasma, and heart plasma from mice fed 0 or 2.5% inulin for 2 weeks. Samples (0 or 2.5% inulin) were compared by unpaired t-test (cecal content) or 2-way ANOVA (sample location × diet) *P < 0.05, **P < 0.01, ***P < 0.001, and ****P < 0.0001.

**Figure 2:**
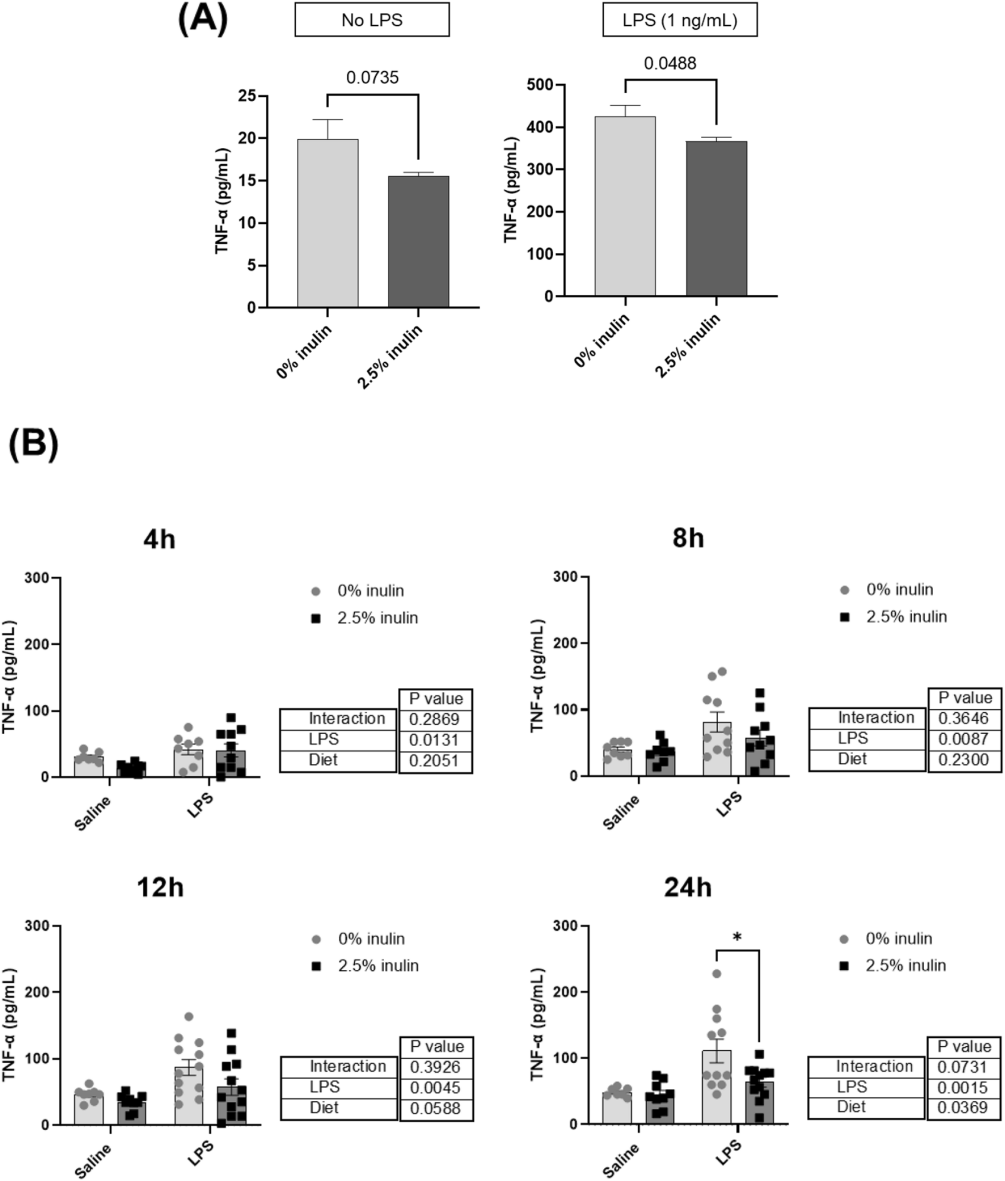
Effect of dietary fiber intake on the TNF-α protein secretion (measured by ELISA) by primary microglia from mice fed 0 or 2.5% inulin for 2 months. TNF-α secretion by microglia was measured (**A**) after isolating and stimulating in vitro with LPS (0 or 1 ng/mL) or (**B**) after injecting mice i.p. with LPS (0 or 500 µg/kg) 4 h before isolating and culturing microglia 4 h, 8 h, 12 h, or 24 h. In (**A**), samples (0 or 2.5% inulin) were compared by unpaired t-test. Statistical difference between inulin levels was considered for *P* < 0.05. In (**B**), samples were analyzed by 2-way ANOVA (2 × 2 factorial arrangement of LPS and diet), followed by Šidák’s multiple comparisons test (*P < 0.05).

In an initial study, mice were fed diet with 0% or 2.5% inulin for 2 weeks and acetate, butyrate, and propionate were measured in cecum and blood plasma collected from the hepatic portal vein or heart (Fig. 1). Dietary fiber increased all three SCFAs in the cecum (*P* < 0.01); and increased butyrate (*P* < 0.0002) and propionate (*P* < 0.0002) in plasma collected from the hepatic portal vein. SCFAs in plasma collected from heart were not affected by diet. In a subsequent study, mice were provided diet with 0% or 2.5% inulin for 8 weeks. Body weight (BW) did not differ between mice fed 0 or 2.5% inulin over the experimental period (20.9 ± 3.0 g BW vs 21.4 ± 3.6 g BW). Microglia were isolated and treated with 0 or 1 ng/mL LPS (Fig. 2). Microglia from mice fed 2.5% inulin tended to secrete less TNF-α in the absence of LPS (*P* = 0.0735) compared to microglia from mice not receiving inulin. When microglia were stimulated with LPS, those isolated from mice fed 2.5% inulin secreted significantly less TNF-α than those isolated from mice not receiving inulin (*P* < 0.05) (Fig. 2A). The intake of fermentable dietary fiber also attenuated microglia inflammation triggered by an in vivo inflammatory insult. Injection of LPS i.p. led to increased ex vivo secretion of TNF-α after a 4, 8, 12, or 24 h culture, compared to microglia from saline-treated mice. When the mice were fed 2.5% inulin and injected i.p with LPS, the ex vivo secretion of TNF-α by microglia after a 24 h culture was significantly lower than that secreted by microglia from mice not fed fermentable dietary fiber and injected i.p. with LPS (*P* < 0.05) (Fig. 2B).

Collectively, these findings suggest that a portion of the SCFAs produced by gut microbiota in response to dietary fiber enters hepatic portal circulation and may regulate microglia activation.

### SCFAs directly inhibit the inflammatory response of stimulated microglia

**Figure 3:**
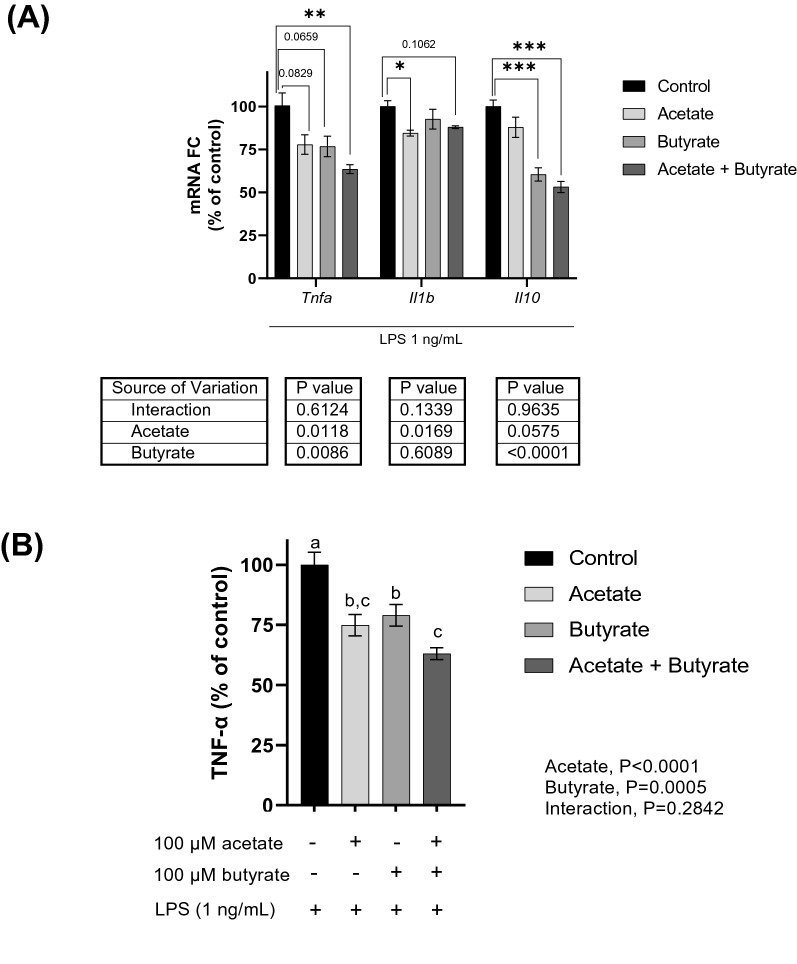
Effect of short-chain fatty acids on (**A**) cytokine mRNA expression (measured by qPCR) and (**B**) TNF-α protein secretion (measured by ELISA) after LPS stimulation of primary microglia. Cells were pre-treated with acetate, butyrate, or combination of both (0 or 100 µM) for 4 h, and then stimulated with LPS (1 ng/mL) for 4 h. Control = cells stimulated with LPS and no pre-treatment with SCFA. Samples were analyzed by 2-way ANOVA (2 × 2 factorial arrangement of acetate x butyrate), followed by (**A**) Dunnet’s test (*P < 0.05, **P < 0.01, ***P < 0.001, and ****P < 0.0001) or (**B**) Tukey’s test. Different lowercase letters are statistically different (*P* < 0.05) among treatments.

To investigate a direct action of SCFAs, microglia were treated with acetate and butyrate, either alone or in combination, prior to LPS stimulation. Stimulation with LPS upregulated *Tnfa*, *Il1b*, and *Il10* mRNA expression in primary microglia. Acetate downregulated *Tnfa* (*P* = 0.012) and *Il1b* (*P* = 0.017), while butyrate downregulated *Tnfa* (*P* = 0.008) and *Il10* (*P* < 0.0001). The combination of both SCFAs led to a 12–47% decrease in the expression of the analyzed cytokines (Fig. 3A). SCFA treatment alone (with no LPS stimulation) did not affect microglia viability (Fig. S1, Supplementary material) or elicited any increase or decrease in mRNA expression.

Supernatant from microglia with no LPS stimulation had TNF-α concentrations below the assay sensitivity (8 pg/mL), while after LPS stimulation the concentration increased to 2513 ± 435 pg/mL. Consistent with gene expression, when pre-treated with either acetate or butyrate, TNF-α protein secretion after LPS treatment was decreased 21–25% (*P* < 0.05). The combination of both acetate and butyrate led to a reduction of 38% (Fig. 3B). The two SCFAs when applied together reduced LPS-stimulated *Tnfa* and TNF-α, in an additive manner. The results indicate acetate and butyrate can act directly on microglia to reduce LPS-induced inflammation.

**Figure 4:**
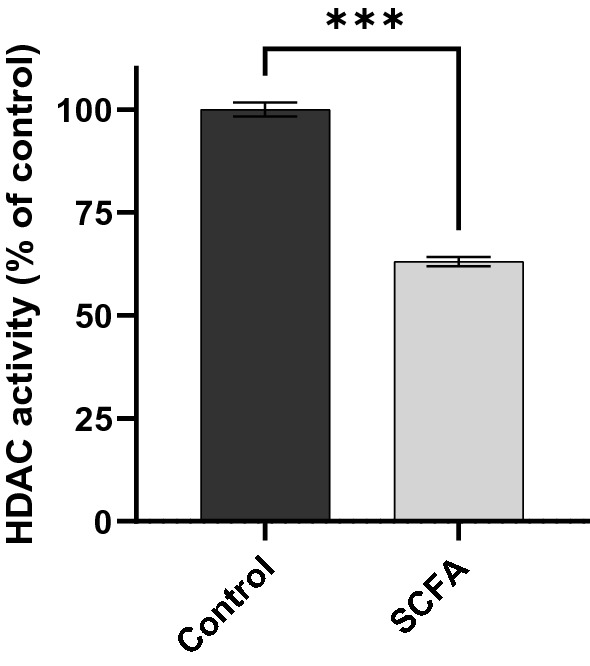
Effect of short-chain fatty acids on HDAC activity in primary microglia after LPS stimulation. Cells were pre-treated with SCFA mix (0 or 100 µM) for 4 h, and then stimulated with LPS (1 ng/mL) for 4 h. Control = cells stimulated with LPS and no pre-treatment with SCFA. Samples were analyzed by t-test with Welch's correction. ***P < 0.001.

**Figure 5:**
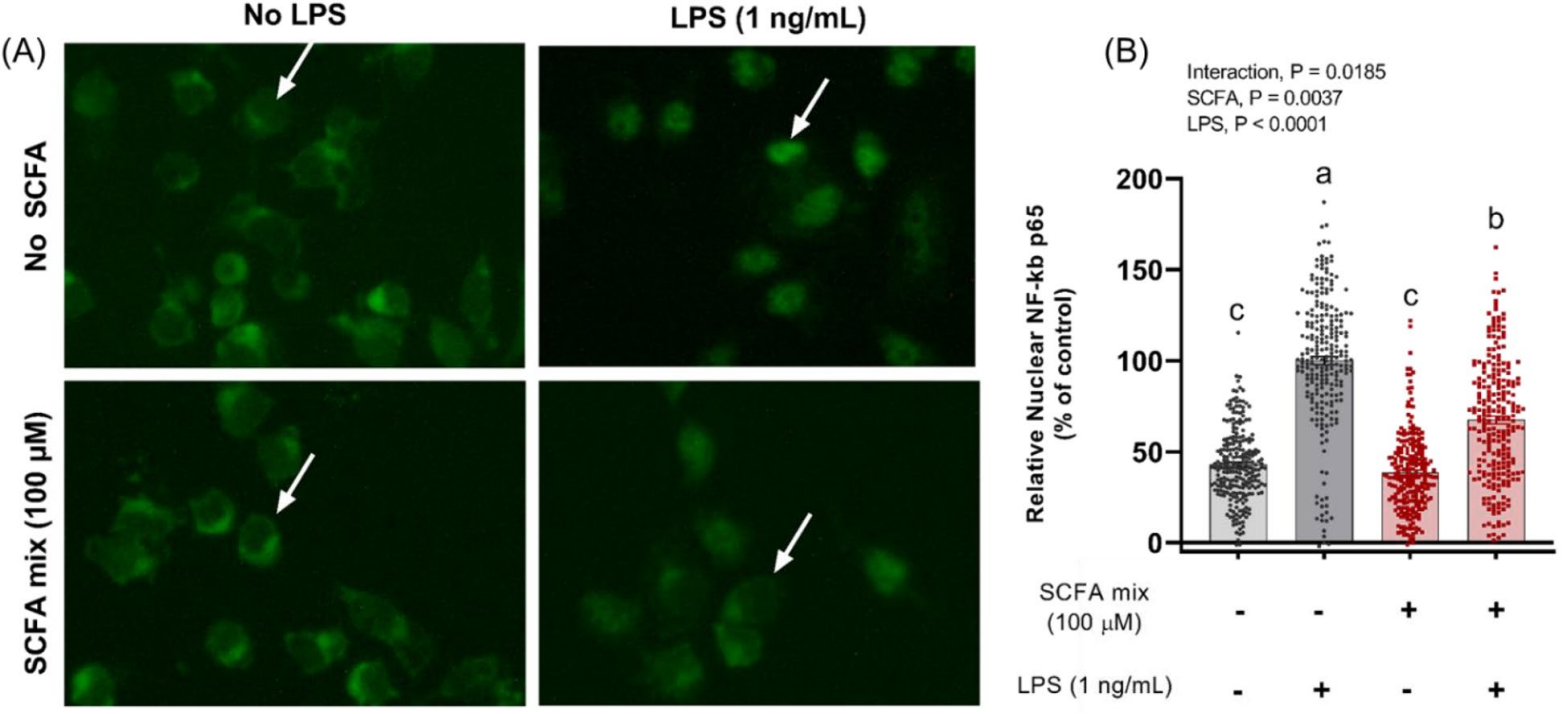
Effect of short-chain fatty acids on nuclear translocation of p65 NF-κB in primary microglia from adult mice 30 min after stimulation with LPS. (Panel **A**) Fluorescence microscopy depicting immunocytochemical localization of NF-κB p65 (green) in primary microglia treated with no SCFA or SCFA mix (100 µM acetate + 100 µM butyrate) with no stimulation (two pictures on the left) and LPS 1 ng/mL stimulation (two pictures on the right). The white arrows point to the nucleus, indicating an example of the intensity of nuclear NF-κB in each treatment. (Panel **B**) Relative Nuclear p65 NF-kB of primary microglia, expressed as percentage of control (cells stimulated with LPS and no SCFA treatment). Primary microglia were plated and pre-treated with 0 or 100 µM acetate and butyrate for 4 h. After 30 min of LPS stimulation (0 or 1 ng/mL), cells were fixed and stained for p65 NF-κB. Values were expressed as the mean of four replicates, whose means resulted from 20 cells from three independent fields of view. Samples were analyzed by 2-way ANOVA (2 × 2 factorial arrangement of SCFA mix x LPS), followed by Šidák multiple comparisons test. Different lowercase letters are statistically different (*P* < 0.05) among treatments.

To investigate the mechanism by which the SCFAs directly inhibit the inflammatory response in microglia, the effect of SCFAs on HDAC activity and NF-κB nuclear translocation were examined. Primary microglia stimulated with LPS showed HDAC activity of 34.8 nmol/min/mg and, when pre-treated with a combination of acetate and butyrate, the HDAC activity was reduced to 22.1 nmol/min/mg, a 36% decrease (Fig. 4). In addition to inhibiting HDAC activity, pre-treatment with SCFAs led to a significant decrease in the LPS-induced nuclear translocation of p65 NF-κB (Fig. 5), indicating the SCFAs inhibit the inflammatory response in microglia by inhibiting NF-κB access to inflammatory gene promotor sites. Apart from histones, HDACs also target transcription-regulating proteins and can activate them by deacetylating lysine residues. Importantly, deacetylation of lysine by HDACs influences the NF-κB pathway and is pro-inflammatory (Leus et al.^24^). Thus, the present results suggest SCFAs inhibit TNF-α production in microglia by reducing HDAC activity, which limits NF-κB activation.

### SCFAs inhibit inflammatory microglia independent of known SCFA receptors and monocarboxylate transporters

**Figure 6:**
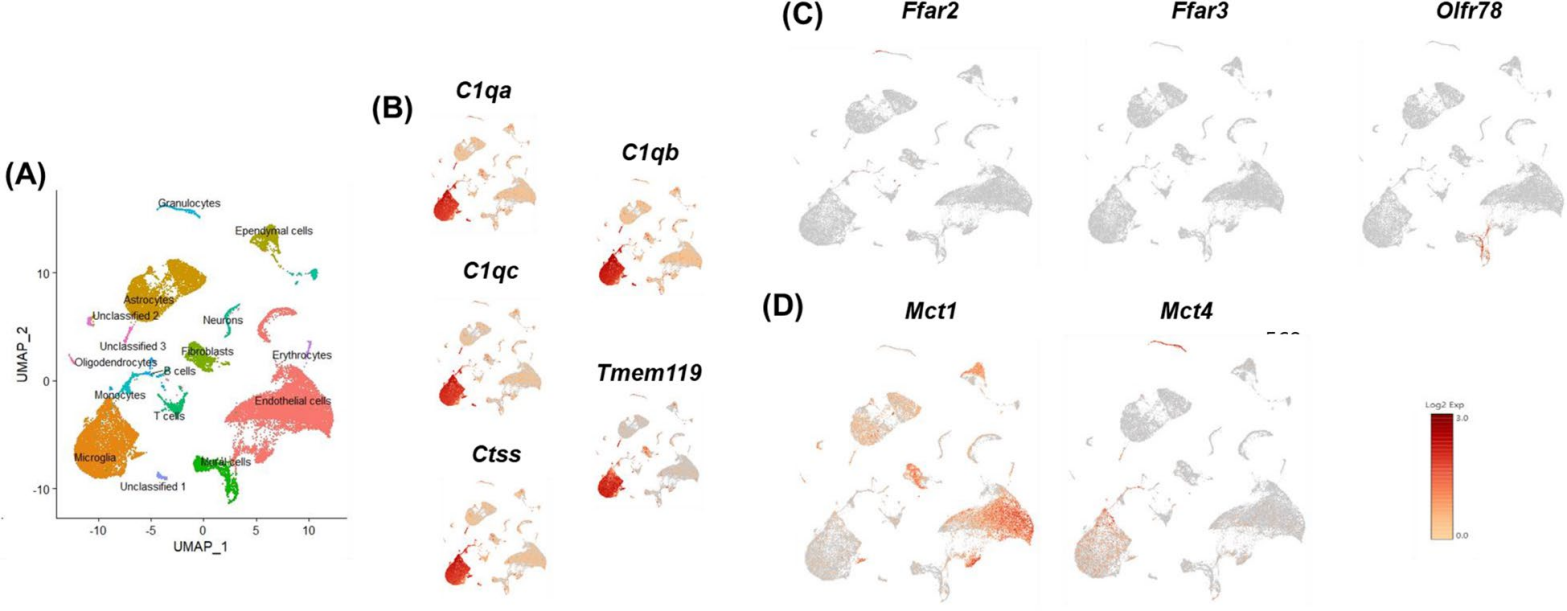
Uniform Manifold Approximation and Projection (UMAP) visualization of cell clusters identified during scRNA-seq analysis of mouse whole brain tissue and SCFA receptors and transporters expression. (Panel **A**) identifies each cell type by name and color. (Panel **B**) illustrates the expression of microglia markers (*C1qa, C1qb, C1qc, Ctss*, and *Tmem 119*) used to identify microglial cells. (Panels **C** and **D**) illustrate the expression of SCFA receptors, *Ffar2, Ffar3*, and *Olfr78*, and transporters, *Mct1 (Slc16a1)* and *Mct4 (Slc16a3*), respectively. Microglia lack the expression of all receptors but express both transporters.

**Figure 7:**
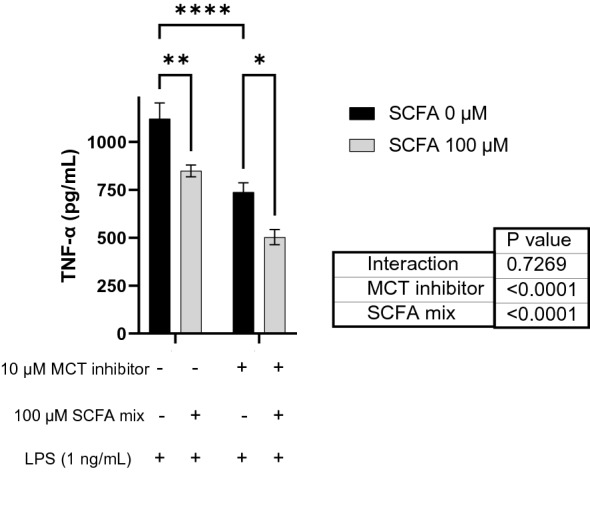
Effect of monocarboxylate transporters (MCT) blockade on short-chain fatty acids activity in primary microglia after LPS stimulation. Cells were pre-treated with SCFA mix (0 or 100 µM) for 4 h, and then stimulated with LPS (1 ng/mL) for 4 h. Samples were analyzed 2-way ANOVA (2 × 2 factorial arrangement of MCT inhibitor x SCFA mix), followed by Tukey's multiple comparisons test. *P < 0.05, **P < 0.01, ***P < 0.001, and ****P < 0.0001.

Using single-cell RNA-sequencing and clustering analysis, we previously revealed 31 clusters in whole mouse brain, which were annotated to 13 unique cell types (Vailati-Riboni et al., 2022). Microglia were identified by expression of *C1qa*, *C1qb*, *C1qc*, *Ctss*, and *Tmem 119*. Here we used Gene-Feature expression analysis to assess microglial cell expression of SCFA receptors *Ffar2*, *Ffar3*, and *Olfr78* as well as the monocarboxylate transporters *Mct1* (*Slc16a1*) and *Mct4* (*Slc16a*). Whereas microglia expression of *Ffar2*, *Ffar3*, or *Olfr78* was not detected, the SCFA transporters *Mct1* and *Mct4* were (Fig. 6). These findings suggest that SCFAs do not inhibit inflammatory gene expression via SCFA receptors, but may do so by a MCT-dependent mechanism. Therefore, to determine the importance of MCTs for the anti-inflammatory effect of SCFAs in microglia, we inhibited MCTs with Syrosingopine prior to treatment with SCFAs and LPS (Fig. 7). Blockade of MCTs alone reduced LPS-induced secretion of TNF-α (*P* < 0.0001). However, blockade of MCTs did not affect the anti-inflammatory action of SCFAs (MCT x SCFA, *P* = 0.7269) indicating SCFAs are not dependent on MCT1 and MCT4. Taken together, these data suggest that similar to epithelial cells^11,12^, SCFAs diffuse across the microglial cell membrane to cause this effect.

## Discussion

The present study shows that feeding a diet with soluble fiber increases SCFAs in cecum and hepatic portal blood and reduces the inflammatory reaction of isolated primary microglia ex vivo. It further shows that SCFAs can act directly on microglia and inhibit HDAC activity, NF-κB activity, and inflammation caused by LPS stimulation. Because the expression of known receptors for SCFAs was not detected in microglia, and blockade of MCTs, which transport SCFAs, did not affect the anti-inflammatory properties of SCFAs, the present results suggest that if SCFAs produced in the gut regulate microglia directly it is likely through an epigenetic mechanism following diffusion.

Microbiota in the cecum and colon ferment soluble fiber to produce bioactive SCFAs. In the present study, mice were fed diet with 1% cellulose, an insoluble fiber resistant to fermentation, or the same diet with 2.5% inulin, a soluble fiber that is readily fermented. As expected, inulin markedly increased acetate, butyrate, and propionate in cecum. SCFAs are a major energy substrate for colonocytes^13^ with butyrate estimated to provide 90% of their energetic requirements. Nevertheless, a portion of the SCFAs reached circulation as butyrate and propionate were elevated in hepatic portal blood of mice receiving inulin. Studies in germ-free mice provide evidence that the gut microbiota and SCFAs regulate microglia. For example, germ-free mice had immature dysfunctional microglia that mostly normalized after colonization with a complex microbiota or administration of a mix of SCFAs via drinking water^14^. In addition, several neurodegenerative diseases that involve microglia activation and neuroinflammation including Parkinson’s and Alzheimer’s diseases, are affected by the gut-brain axis^15,16^. The present study extends earlier reports by showing that a diet that increases SCFAs inhibits inflammation in microglia. This is important since up to 40% of adults in the U.S. consume less fiber than recommended^17^.

Circulating SCFAs enter the brain. Acetate, butyrate, and propionate are detectable in human cerebrospinal fluid^18^. Furthermore, the levels of butyrate in the brain of mice supplemented with *Clostridium butyricum*, a butyrate-producing probiotic, were elevated^8^. It is noteworthy that the butyrate concentration measured in the brain was an order of magnitude higher than that measured in peripheral blood, an issue attributed to the abundant expression of MCTs in endothelial cells that form the blood–brain barrier^19^. Finally, acetate supplementation led to higher brain acetate levels in rats^9^. Acetate, the most abundant SCFA in brain of mice, is reduced in germ-free mice^20^. In the present study, if SCFAs concentrated in brain, it could explain why primary microglia from mice fed inulin for 8 weeks were less responsive to either ex vivo LPS stimulation or LPS i.p. injection. This is one reason we examined the anti-inflammatory mechanisms of SCFAs on microglia in vitro.

Treatment with acetate, butyrate, or the combination of the two inhibited HDAC activity, NF-κB activity, *Tnf* expression and TNF-α secretion after LPS treatment. These results were observed even though the expression of SCFA receptors was not detected in microglia by our scRNA-Seq analysis, nor by others using the same analysis^21^ or by qPCR^14^. The anti-inflammatory effects of acetate and butyrate were additive. Despite microglia expressing MCTs, the inhibition occurred independent of MCTs since treatment with Syrosingopine did not interfere with the anti-inflammatory effects of the combination of acetate and butyrate. Our interpretation is that SCFAs diffused across cell membranes to inhibit inflammation. Posttranslational histone modification is controlled by the opposing actions of histone acetyltransferases and HDACs. By removing acetyl groups, HDACs alter gene transcription by condensing chromatin. When acting via FFAR2 on neutrophils and monocytes, SCFAs can be pro-inflammatory^6^. However, most receptor-independent effects of SCFAs are anti-inflammatory and mediated by inhibition of HDACs^22^. Our finding that butyrate and acetate reduced HDAC activity and inflammation in microglia, which do not express FFAR2, is consistent with this progression of thinking.

In addition to affecting HDAC activity, SCFA treatment decreased nuclear p65 NF-κB following LPS stimulation. Apart from deacetylating histones, HDACs deacetylate lysine residues of NF-κB subunits and thereby regulate nuclear translocation, DNA-binding, and overall transcriptional activity^23^. The fluorometric assay used herein measured Class I and Class II HDAC activity in nuclear extracts. This includes HDAC 3, which is an activator of NF-κB-mediated inflammatory gene expression due to its ability to deacetylate four specific lysine residues (K314, K315, K122, K123) of p65 NF-κB and keep it in an active (deacetylated) state^24^. This is the only HDAC that deacetylates p65. HDAC 3 is downregulated in microglia following treatment with acetate, as is the expression of IL-1β, IL-6, and TNF-α after LPS-stimulation^23^. Since acetylated p65 accumulates in the cytoplasm, its reasonable to postulate that the decreased nuclear translocation of p65 observed in the present study is due to inhibition of HDAC activity and acetylation. Future studies where SCFAs are increased by dietary fiber and epigenetic pathways are assessed in primary microglia will help understand better the mechanisms of the gut-brain axis.

While we show that a diet that increases SCFAs regulates microglia and that direct treatment of microglia with SCFAs inhibits inflammatory response, we do not discount other indirect mechanisms. For example, aged mice fed a low fiber diet that reduced SCFAs in cecum, had increased inflammatory infiltrate in the colon (Matt et al., 2018). While it is uncertain if this was directly due to changes in SCFAs, conditions that increase the peripheral inflammatory tone tend to increase neuroinflammation as inflammatory cytokines convey information to the brain to regulate microglia^25,26^. Immunoregulation is predictable since neutrophils and regulatory T cells of the mucosal immune system express SCFA receptors^6,27^. Indeed, stimulation of FFAR2 by SCFAs was necessary to resolve inflammation in models of colitis, arthritis, and asthma^6,28^. In cases of unresolved peripheral inflammation, it is reasonable to expect microglia activation due to the pathways connecting the peripheral immune system and brain. It is also noteworthy that receptors for SCFAs are present on vagal afferent nerves that innervate the hepatic portal vein where we found elevated levels of butyrate and propionate in mice fed inulin. Therefore, it remains an open question if SCFAs affect microglia through neural and/or humoral pathways.

## Methods

### Animals

Adult (2–4 month old) male and female C57BL/6 J mice from our in-house breeding colony were used. They were kept in the same room under a reverse 12 h light:dark cycle and given ad libitum access to water and diet. Investigators could not be blinded to the mouse diet due to the difference in diet colors, but they were blinded for all the analyses performed after euthanasia. All animal care and handling procedures were carried out in accordance with NIH and ARRIVE guidelines and approved by the University of Illinois Institutional Animal Care and Use Committee.

**Figure 8:**
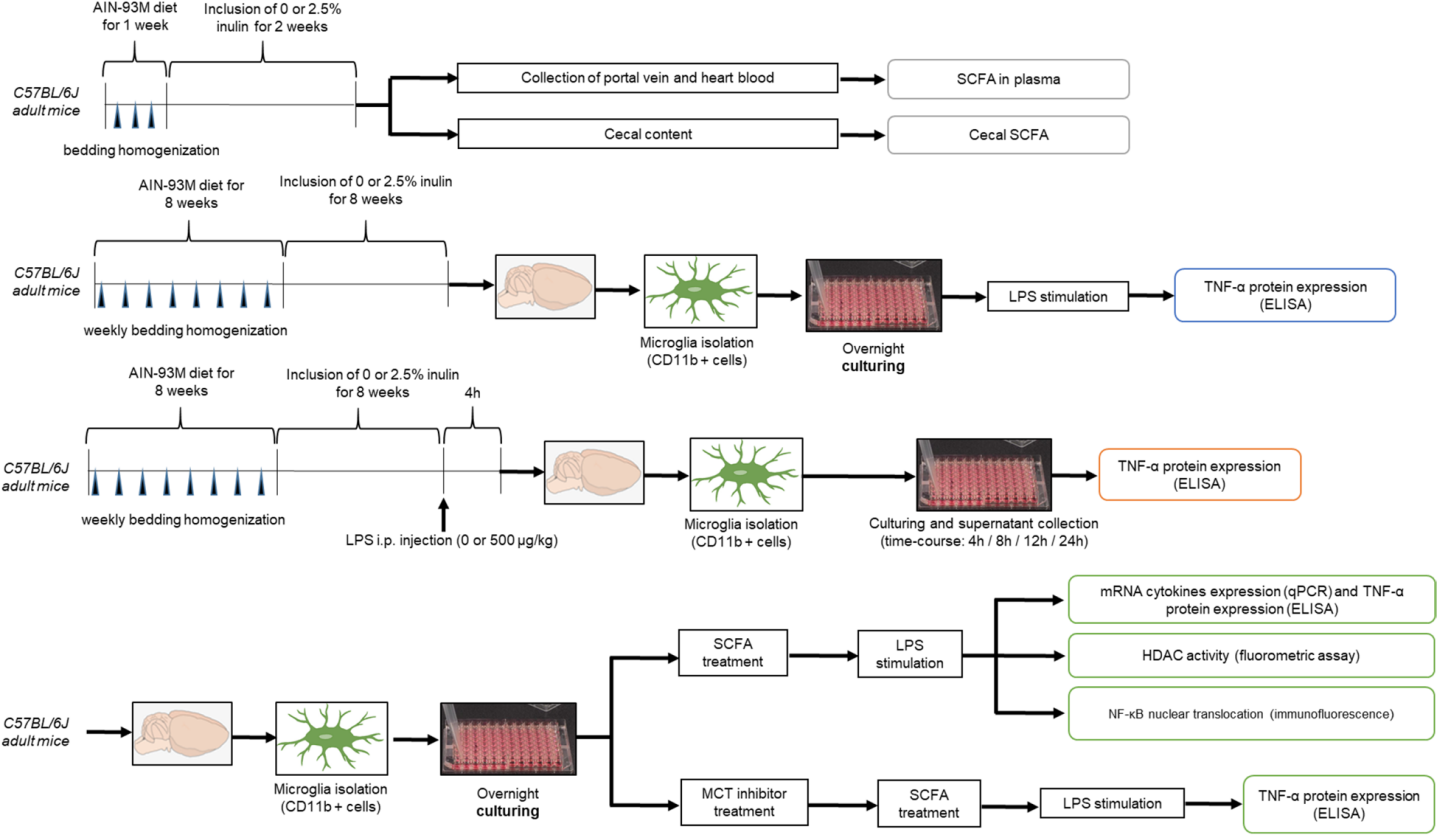
Experimental design. Specific conditions such as time and concentrations are described in the Methods section and in the respective Figure legend.

Figure 8 shows the experimental design for the study performed.

### Primary microglia isolation

Mice were euthanized by CO_2_ asphyxiation and transcardially perfused with sterile ice-cold PBS. The whole brain was dissected and microglia were isolated using the Neural Tissue Dissociation Kit (P) (Miltenyi Biotec, Germany) and CD11b magnetic beads, according to procedures previously described by Nikodemova and Watters^29^. Briefly, whole brain was enzymatically digested at 37 °C for 22 min. Tissue debris and myelin were removed by passing the cell suspension through a 70-µm cell strainer and then resuspending in 30% Percoll Plus (GE Healthcare, Princeton, NJ, USA) and centrifuging (300 × g/8 min). Cells were incubated with anti-CD11b magnetic beads (Miltenyi Biotec, Germany) for 22 min at 4 °C. CD11b^+^ cells were separated in a magnetic field using MS columns (Miltenyi Biotec, Germany) after washing with PEB buffer (PBS supplemented with 0.5% BSA and 2 mM EDTA). Microglial cells were resuspended in Dulbecco's Modified Eagle medium (DMEM) supplemented with 10% FBS and penicillin (100 U/ml) and streptomycin (100 μg/ml) at 37 °C and plated as described below for experiments. In all cases, cell viability was confirmed by MTT and LDH (Sigma Aldrich, MO, USA, Cat #M6494 and #11644793001) assays, according to manufacturer's instructions, to ensure treatments were not toxic.

## Experiments

### Effect of dietary fiber on plasma and cecal SCFA levels

Mice had ad libitum access to AIN-93 M diet (Envigo, Indianapolis, IN) for one week and modified AIN-93 M diet with 0 or 2.5% inulin for two weeks. Four hours after the onset of darkness, mice (n = 11) were anesthesized with isofluorane and blood from the hepatic portal vein and heart was collected using a 30 ga. needle and Li heparin syringe before mice were euthanized by CO_2_ asphyxiation. Samples were centrifuged 10 min at 2000 g to obtain plasma, which was diluted 1:1:1 with acetonitrile and meta-phosphoric acid (6.25%), and then frozen at -80°C until SCFA analysis. Cecal contents were collected and diluted 1:4 with meta-phosphoric acid (6.25%). An aliquot of the cecal content was weighed and used for dry matter (DM) analysis. Cecal samples were frozen at −20 °C until SCFA analysis.

Short-chain fatty acids were analyzed by gas chromatography coupled with mass spectrometry using an Agilent 7890 (Agilent Inc., Palo Alto, CA, United States) gas chromatograph with an Agilent 5975 mass selective detector and Agilent 7683B autosampler. The column used was a 30 m HP-INNOWAX (0.25 mm inner diameter and 0.25 µm film thickness, Agilent, Palo Alto, CA, United States). Sample volume and flow rate of Helium were set for 1 µL and 1 mL/min, respectively. The temperature was 200 °C for injection and MSD transfer line, and 230 °C for the ion source. The temperature program was as follows: 2 min at 70 °C, oven temperature ramp of 10 °C/min to 190 °C and 40 °C to 240 °C for a final 2 min. Standard curves of SCFAs were used for SCFA measurement in the range 0.1–50 mg/L.

Mass spectrometry settings were: positive electron impact mode (EI) at 69.9 eV ionization energy in m/z 30–300 scan range in combined scan and selected ion monitoring (SIM) modes., and SIM targeted m/z 43, 45, 46, 60, 74. The software Mass Hunter Quantitative Analysis B.08.00 (Agilent Inc., United States) was used for the evaluation of target peaks. Concentrations of acetate, butyrate, and propionate were expressed as µmol/g of DM for cecal contents and as mM for portal vein and heart blood.

### Effect of dietary fiber on microglia

Two procedures were adopted in order to bring gut microbiota into conformity, and thus reduce variability and increase reproducibility in the experiments^30^: (1) mice had ad libitum access to AIN-93 M diet for eight weeks; and (2) during weekly cage changes, used bedding (i.e., litter and feces) was homogenized, mixed with clean bedding, and redistributed to each cage. Body weights (BW) were recorded weekly. Following the acclimation period, mice had ad libitum access to a modified AIN-93 M diet containing 1% cellulose and either 0 or 2.5% inulin (Envigo, Indianapolis, IN) for eight weeks.

In one experiment, microglia from 2–3 mice fed 0 or 2.5% inulin were pooled (twelve mice in total), allocated to 96-well plates at 1 × 10^5^ cells/well (200 µL/well), and incubated at 37 °C in a humidified incubator under 5% CO_2_. Microglia were allowed to adhere overnight and then treated with *Escherichia coli* lipopolysaccharide (LPS; O127:B8; Sigma Aldrich, MO, USA) (0 or 1 ng/mL) for 24 h. The supernatant was collected and stored at −80°C until TNF-α analysis by ELISA.

In another study, 40 mice were intraperitoneally (i.p) injected with 0 or 500 µg LPS/kg BW. Body weight was recorded before and 4 h afterLPS injection when mice were killed. Microglia were isolated and cultured in 10% FBS DMEM as described above. The supernatants were collected at 4, 8, 12, and 24 h after plating and stored at -80°C until TNF-α analysis by ELISA.

### SCFA treatment of primary microglia and LPS stimulation

Primary microglia isolated from mice maintained on standard rodent chow were used. Microglia from 18 to 24 brains were pooled and allocated to 96-well plates at 1 × 10^5^ cells/well (200 µL/ well) or in 24-well plates at 5 × 10^5^ cells/well (800 µL/well), depending on the subsequent analysis to be performed and the quantity of cells required. Microglia were incubated at 37 °C in a humidified incubator under 5% CO_2_ and allowed to adhere overnight. Cells were treated with sodium acetate, sodium butyrate (Sigma Aldrich, MO, USA, Cat #S5636 and #B5887) (0 or 100 µM), or the combination of both SCFAs for 4 h, and then treated with LPS (0 or 1 ng/mL) for 4 h (n = 9–12).

*Cytokine expression.* After LPS treatment, supernatant was collected and microglia were resuspended in Trizol reagent (Invitrogen, Carlsbad, CA, USA). Both supernatant and cells were stored at −80 °C until further analysis. The concentration of TNF-α protein in the conditioned supernatants was assessed using a commercially available ELISA kit (Thermo Fisher, Waltham, MA, USA) according to manufacturer's instructions. Total RNA was extracted from microglia using a DirectZol kit (Zymo Research), following manufacturer's protocols. RNA concentration was adjusted to 40 ng/µL and cDNA synthesis was performed using the High-Capacity cDNA Reverse Transcription Kit (Thermo Fisher, Waltham, MA, USA), including Oligo dT18 (Integrated DNA Technologies, Coralville, IA, USA). *Gapdh* (Glyceraldehyde 3-phosphate dehydrogenase) (Mm.PT.39a.1 PrimeTime qPCR Assay, Integrated DNA Technologies) was selected as the internal control. The target genes analyzed were *Tnf* (Mm.PT.58.29509614), *Il-1b* (Mm.PT.58.41616450), and *Il-10* (Mm.PT.58.23604055). The polymerase manufacturer recommended thermal cycle was used in a QuantStudio 7 real time PCR machine (Thermo Fisher), and the calculations were done using the relative standard curve method (generated from a pool of all cDNA pre-dilution). Target gene relative quantities were normalized using the internal control gene relative, and log2 transformed prior to statistical analysis.

*HDAC activity.* After SCFA treatment and LPS stimulation as described above, microglial cells were collected in ice-cold hypotonic solution supplemented with phosphatase inhibitors, and nuclear extracts were obtained using a nuclear extraction kit (Cayman Chemicals, Cat #10009277) following the manufacturer's recommendations. For each treatment, cells from 12 wells (from a 24-well plate) were pooled. Class I and II HDAC activity was measured using a fluorometric assay (Cayman Chemicals, Cat #10011563). The method is based on the deacetylation of an acetylated lysine substrate by the HDAC contained in the nuclear extracts. Deacetylation sensitizes the substrate such that treatment with the HDAC developer reagent releases a fluorescent product. The fluorophore was analyzed using a fluorescence plate reader with excitation and emission wavelengths of 360 nm and 460 nm, respectively. HDAC activity was normalized by protein content in the nuclear extracts, determined by Bicinchoninic acid assay (Thermo Fisher, Waltham, MA, USA, Cat #23225), according to manufacturer’s instructions and then expressed as percentage of control (LPS-stimulated primary microglia with no SCFA treatment).

*NF-κB nuclear translocation.* After SCFA treatment as described above, microglia were treated with LPS (0 or 1 ng/mL) for 30 min for maximal translocation of NF-κB p65 into the nucleus^31^. Cells were washed with PBS and fixed with 4% paraformaldehyde (PFA) for 10 min. After PFA removal and 3 washes with PBS, cells were blocked with 5% goat serum in 1 × PBS 0.3% Tween for 1 h, and then incubated overnight at 4 °C with primary antibody NF-κB p65 (D14E12) XP Rabbit mAb (Cell Signaling Technology, Inc., Danvers, MA, USA) (dilution 1:4000 in 1X PBS/1% BSA/0.3% Triton X-100). After incubation with secondary antibody (AlexaFluor 488 Goat anti-Rabbit IgG (H + L) (dilution 1:1000) for 1 h, nuclei were stained with Hoechst (Life Technologies, Cat #H3570, 10 mg mL^−1^, Eugene, OR, USA) for 5 min protected from light. An inverted microscope with an epifluorescence (Laxco LMI 6-FL88, 40X (NA 0.65) objective, and 5.1MP CMOS camera) was used to acquire the fluorescent images. The fluorescence intensity in the nucleus of the stained microglia was determined using the ImageJ software (U. S. National Institutes of Health, Bethesda, MD, USA, https://imagej.nih.gov/ij/, 1997–2018) and used to calculate the nuclear translocation of NF-κB p65 as a percentage of level measured in control (cells not treated with SCFAs but stimulated with LPS).

*Inhibition of monocarboxylate transporters (MCT).* In a pilot study, microglia were treated with 0, 100, 500, 1000, 5000, or 10,000 nM of the MCT inhibitor, Syrosingopine (Sigma Aldrich, MO, USA, Cat #SML1908), to identify a concentration reported to block MCT^32^ and that was not lethal for microglia. In a subsequent study, microglia were treated with 0 or 10,000 nM Syrosingopine for 3 h; then SCFAs (acetate and butyrate as described above) for 4 h; followed by LPS (0 or 1 ng/mL) for 4 h. TNF-α protein was measured in conditioned supernatants by ELISA.

*Whole brain single-cell RNA sequencing (scRNA-seq) analysis.* Tissue dissociation, library preparation, sequencing, and data processing methods were previously described by Vailati-Riboni, Rund^30^. The single-cell 3′ cDNA libraries were prepared by the DNA Services Laboratory of the Roy J. Carver Biotechnology Center at the University of Illinois at Urbana-Champaign. The clusters for cell type identification were based on data previously described ^30^. The expression of the microglia markers (*C1qa, C1qb, C1qc, Ctss*, and *Tmem 119*), SCFA receptors (*Ffar2, Ffar3*, and *Olfr78*), and MCTs [*Mct1 (Slc16a1)* and *Mct4 (Slc16a3*)] by microglia was analyzed using the software Loupe Browser 6.1.0 and presented as Uniform Manifold Approximation and Projection (UMAP) for the purpose of data visualization.

### Statistical analysis

Statistical analyses were performed using the statistical package GraphPad Prism 8.0.1 (GraphPad Software, Inc., San Diego, CA, USA). Data were subjected to one- or two- 2-way ANOVA followed by Dunnett's test. The graphs present the statistical significance following the convention *P < 0.05, **P < 0.01, ***P < 0.001, and ****P < 0.0001. For TNF-α protein secretion and NF-κB nuclear translocation analyses, Tukey's test for multiple comparisons between each treatment was performed. Different letters represent statistical difference between samples.

## Supplementary Information

Supplementary Information.

## Data Availability

The sequencing data have been submitted to Gene Expression Omnibus database (GEO Accession: GSE163055.
