# Supplementary material for: Inhibition of inflammatory microglia by dietary fiber and short-chain fatty acids
Source: Sci Rep. 2023 Feb 16;13:2819. doi: 10.1038/s41598-022-27086-x (PMC9935636; doi:10.1038/s41598-022-27086-x)
Supplement: Supplementary file 1 — Supplementary Information. [file 41598_2022_27086_MOESM1_ESM.docx]

**Supplementary material**

**
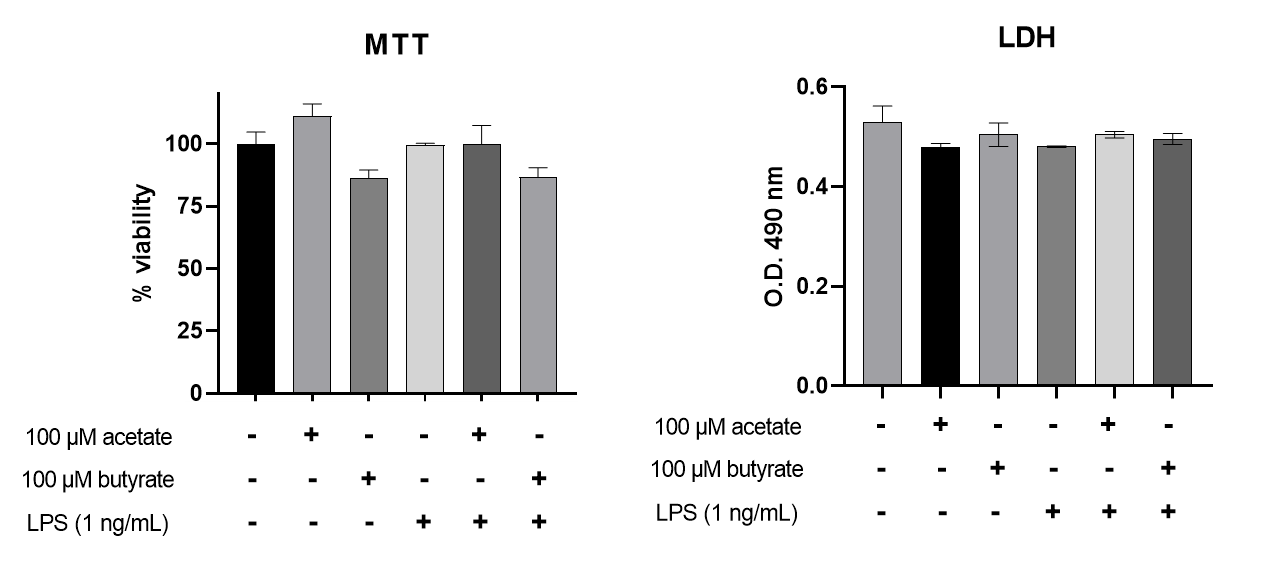
**

**Figure S1.** Cell viability assessed by (A) MTT and (B) LDH assays after SCFAs treatment (0 or 100 µM) and LPS stimulation (0 or 1 ng/mL). MTT and LDH (Sigma Aldrich, MO, USA, Cat #M6494 and #11644793001) assays were performed according to manufacturer's instructions.
